# Prevalence and Associated Factors of Tuberculosis among Adult Household Contacts of Smear Positive Pulmonary Tuberculosis Patients Treated in Public Health Facilities of Haramaya District, Oromia Region, Eastern Ethiopia

**DOI:** 10.1155/2020/6738532

**Published:** 2020-01-27

**Authors:** Abinet Adane, Melake Damena, Fitsum Weldegebreal, Hussein Mohammed

**Affiliations:** ^1^East Hararghe Zone, East Hararghe Zonal Health Office, Harar, Ethiopia; ^2^School of Public Health, College of Health and Medical Sciences, Haramaya University, P.O. Box: 235, Harar, Ethiopia; ^3^Department of Medical Laboratory Sciences, College of Health and Medical Sciences, Haramaya University, Harar, Ethiopia

## Abstract

**Background:**

Tuberculosis is an infectious airborne disease caused by *Mycobacterium tuberculosis*. It still remains a major public health problem which affects all age groups. Risk of exposure is higher in household contact than members of the general population.

**Objective:**

The aim of this study was to assess the prevalence and associated factors of tuberculosis among adult household contacts of smear positive pulmonary tuberculosis in Haramaya district, Oromia Region, Eastern Ethiopia from February to March, 2019.

**Method:**

A community based cross-sectional study design was conducted. A total of 454 study participants were selected using systematic sampling method from all adult household contacts of smear positive pulmonary tuberculosis patients treated from July 2017 to December 2018. Data were collected using a pretested and structured questionnaire; and laboratory examination was processed using fluorescent smear microscope. Logistic regression analysis was used to identify the factors associated with the infection of pulmonary tuberculosis and a statistically significant association was declared at *P*-value < 0.05.

**Result:**

The overall prevalence of pulmonary tuberculosis among adult household contacts was 7.8% (95% CI: 5.8–10.0). The risk factors for tuberculosis infection among household contacts were eating meals less than three times per day (AOR = 4.31; 95% CI: 1.61, 11.55), drinking raw milk (AOR = 4.12; 95% CI: 1.43, 11.90), having family history of tuberculosis with more than one index case (AOR = 2.7; 95% CI: 1.02, 6.92), living in poor ventilated houses (AOR = 4.02; 95% CI: 1.38, 11.76), and living in inadequate size of living room (AOR = 3.4; 95% CI: 1.30, 8.86).

**Conclusion:**

In this study, the prevalence of tuberculosis among adult household contacts of smear positive pulmonary tuberculosis is high. Eating meals less than three times per day, drinking raw milk, living in poor ventilated houses, and inadequate sizes of the rooms were identified as contributing factors. Therefore, we recommend that the transmission of tuberculosis can potentially be reduced by a better contact tracing and treatment strategies along with appropriate health education.

## 1. Introduction

Pulmonary tuberculosis (PTB) is an infectious airborne disease caused mainly by *Mycobacterium tuberculosis*. The main source of infection is untreated smear-positive PTB patients. It typically affects the lungs (PTB) but can also affect other sites as well (extra PTB) [1]. The chance of developing TB is much higher among people with low immune status [2, 3]. Tuberculosis (TB) disease remains a major public health problem which affects all age groups globally [1]. It is one of the top 10 causes of death and the leading cause from a single infectious agent and millions of people continue to fall sick with TB each year [2].

The global TB report showed that there were an estimated 10 million incident cases and 12 million prevalent cases of TB globally. Overall 90% of the infection occurred in adults; of this 9% were people living with HIV (72% in Africa). About 26% of incident TB cases occurred in Africa and 23% of the world's population are estimated to have a latent TB infection, and are thus at risk of developing active TB during their lifetime [2]. Currently, Ethiopia is ranked eighth among the 22 high TB burden countries in the world and at rank three, in Africa. The incidence rate of all forms of TB is estimated at 164 per 100,000 population, leading to an annual mortality rate of 27.5 per 100,000 population [1, 2].

Tuberculosis is exclusively transmitted based on environmental and personal risk factors, especially in a social mixing setting (together with overcrowding) and conditions which prolong the length of exposure to an infectious patient like health system-related factor including delay in diagnosis can increase TB transmission [4, 5]. In addition, the risk of infection following TB exposure is primarily governed by exogenous factors and intrinsic combination of the infectiousness of the source case, proximity to contact and social and behavioral risk factors including smoking, alcohol, and indoor air pollution [4].

Patients with asymptomatic pulmonary tuberculosis transmit the bacilli to risky groups through inhalation. Identify and investigating infected individual among contacts of patients with infectious tuberculosis is the best method of preventing the later development of the disease in populations. In addition to this, contact tracing is very important to establish the primary source of the TB disease and to detect those who are secondarily infected by proper diagnosis and prompt treatment [6–8]. Furthermore, the likelihood of progression from latent TB infection (LTBI) to TB in household contacts is usually higher than in the general population. Household contacts of PTB patients are considered a high-priority population for contact investigation [9–11].

Pulmonary tuberculosis contact investigations are rarely and inconsistently carried out in resource-limited settings of low- and middle-income countries like Ethiopia [12, 13]. Studies conducted in Ethiopia on the prevalence and associated factors of PTB among household contacts were very limited. So, it is necessary to identify the factors which contribute to TB infection and overall status of PTB among household contacts of smears positive TB in the district.

## 2. Methods and Materials

### 2.1. Study Area and Period

The study was conducted in Haramaya district, Oromia region, eastern Ethiopia from February to March, 2019. Haramaya is one of 24 districts in east Hararghe zone which is located at 520 km from Addis Ababa; capital city of Ethiopia, and 20 km from the historical city, Harar, with a total land area of 525.64 sq km. It is bordered by Kurfachelle district in north, Dire-Dawa administration in south, Kersa and Kombolcha districts in west and east, respectively. According to population projection set in 2018, the total population of the district is 304,276 among which 152,442 (50.1%) were males and 151,834 (49.9%) were females. Administratively, the district has 2 urban and 31 rural kebeles, with one district hospital, eight health centers, 12 private clinics, and 33 healthposts. All governmental health facilities are providing diagnostic and treatment services for tuberculosis [14].

### 2.2. Study Design and Population

A community based cross-sectional study design was employed. Adults who had close household contact with PTB infected patients who were on treatment from July 2017 to December 2018 were included while critically sick and mentally ill were excluded from the study.

### 2.3. Sample Size Determination and Sampling Procedure

The sample size was computed using a single population proportion formula by considering 4.3% proportion of TB cases among household contacts of smear-positive PTB (1), 95% confidence interval, and 2% margin of error. Accordingly, the final sample size with a 5% non-response rate was 454. All public health facilities (one hospital and eight health centers), providing TB diagnosis and treatment services in the district were considered for the study. According to the information retrieved from these health facilities, a total of 240 TB patients were registered and treated from July 2017 to December 2018. First, the total adult household contacts of smear-positive PTB patients (*n* = 1152) were obtained from the district's Community Health Information System registries available at health post. Then the sample size was proportionally allocated to each health facility according to the number of adult household contacts of smear positive PTB patient. Finally, 454 study participants were randomly selected from households.

### 2.4. Data Collection Tools and Methods

Data were collected using a structured questionnaire developed from previous literature [15–17] and it was pretested on 5% of the study participants in a nearby district (i.e., Aweday district). Data collectors and supervisors were trained on data collection tools and how to observe measure, collect, and transport samples to health facility that helped minimize bias, and maintain the quality of data. Moreover, the supervisors and principal investigator have been supervising field works and they checked the quality of collected data on a daily basis.

The data were collected through face to face interview, observing the house condition, measuring area of house, and collecting data from adult household contacts. Interview and observation were conducted prior to sputum sample collection after informing; getting consents and screen household contacts by using symptoms based algorithm. Household contacts were traced by home to home visits. Sputum microscopy was used to determine tuberculosis among adult household contacts. Household contacts were requested to submit two sputum specimens. Sputum collected at household level was transported to health facility using triple packaging by maintaining cold chain within 5 days. Participants were advised to submit sputum not containing food particle and minimize amount of saliva. Sputum microscopy was done in Haramaya district hospital laboratory using fluorochrome staining technique. In this study Presumptive TB cases were diagnosed if an individual had one or more of the clinical manifestations consistent with TB (i.e. persistent cough of 2 weeks or more, fever for more than 2 weeks, night sweats, and unexplained weight loss) and TB patients were diagnosed if participants had at least one sputum smear positive (1).

### 2.5. Data Quality Control

Data collectors and supervisors were trained for two days on the data collection tools. The collected data were checked manually on daily basis to check for its completeness and consistency. Double data entry were done by two separate individuals to crosscheck the data entered. The recommended procedure for specimen collection, proper labeling, and storage was followed strictly. The sample was rejected if it contained food particles. Internal and external quality control was also ensured for AFB smear microscopy.

### 2.6. Data Processing and Analysis

The data were coded and double entered to Epi data version 3.1 and then exported to Statistical Package for Social Sciences (SPSS) version 21 for analysis. Data cleaning was performed by running frequencies and cross tabulation to check accuracy, outliers, consistencies, and missing values. Descriptive statistics (i.e. frequency distribution, proportion, mean, and standard deviation) was used to summarize the variables. Bivariate analysis was performed to assess the potential associations between categorical variables and the outcome. Variables with a *p*-value of less than 0.25 in the bivariate analysis were entered into the final multivariable logistic regression model to identify the predictors of the outcome. A variable with a *P*-value less than 0.05 at 95% confidence level was considered statistically significant. The absence of multi-co-linearity was checked by using VIF/tolerance. The model of fitness was checked by Hosmer and Lemeshow, which provided evidence of fitness with a predictor test level of *p* = 0.82.

## 3. Results

### 3.1. Socio-Demographic Characteristics

A total of 240 index cases registered in selected public health facilities in the district were included in the study. More than half of the index cases 122 (50.8%) were male and most of them (80%) were from rural areas. From a total of 454 planned household contacts, 451 were included in the study, with a response rate of 99.3%. The mean (±SD) age of the household contacts patients was 31.0 (±11.7) years, range from 18 to 75 years. More than half, 228 (50.6%), of the household contacts were female, majority (73.8%) were from rural areas, and 60.1% were married. One hundred seventy six (39%) of household contacts' relationships with index cases were siblings followed by husband (24.8%) and wife (23.3%). Three hundred thirty eight (74.9%) of the household contacts were farmer in occupation (Table 1).

### 3.2. Housing Condition of the Participants

Four hundred sixteen (92.7%) of houses of the study participants were made from mud and wood. Majority 305 (67.6%) of the household contact houses had good ventilation and 314 (69.6%) of the houses had good lighting status. Three hundred sixty sixes (81.2%) of the respondents had separate kitchen room and 408 (90.5%) of household contacts used wood for cooking food (Table 2).

One hundred nine (24.2%), 206 (45.7%), 104 (23.1%), and 12 (2.7%) of household contacts had habit of cigarette smoking, khat chewing, raw milk consuming and alcohol drinking, respectively. Majority (86.3%) of the household contacts had a habit of eating meals ≥3 times per day. Two third (67.6%) of study participants had no family history of TB (Table 3). 362 (80.3%) of household contacts had adequate knowledge about TB.

### 3.3. Prevalence of Pulmonary Tuberculosis

The overall prevalence of tuberculosis among adult household contacts was 35 (7.8%) (95% CI: 5.8–10). Among household contacts, 206 (45.6%) were symptomatic (had cough > 2 week), and of these 91 (44%) had chest pain, night sweating, and fever. The proportion of tuberculosis among presumptive TB cases was 35 (16.9%). Among detected tuberculosis cases, a relatively higher proportion was observed among age group of 28–37 years (48.6%), male (57.1%), married (71.4%), farmer (85.7%), having monthly average income of <1000 Birr (82.9%), and illiterate (74.3%) adult household contacts (Table 4).

### 3.4. Associated Factors

In bivariable analyses, age of the adult household contacts, income, ventilation status of houses, lighting status of houses, overcrowdings, presence of separate kitchen, the size of living room, proximity to index case, treatment duration of TB index case, family history of TB, smoking cigarette, chewing khat, drinking raw milk, and frequency of meals per day on TB were found to be associated with PTB at *p*-values less than 0.25.

In multivariable analysis frequency of meals per day, house ventilation status, living room size, drinking raw milk, and family history of TB other than index case remained statistically significant at *p*-value less than 0.05. Study participants eating meals less than three times per day were 4 times more likely to acquire TB (AOR = 4.31; 95% CI: 1.617, 11.545) as compared to those eating meals more than three times per day. Study participants who had history of drinking raw milk were about 4 times more likely to develop tuberculosis (AOR = 4.12; 95% CI: 1.43, 11.90) than their counterparts. The odds of getting TB was about 3 times higher among household contacts who had history of TB within family other than index case (AOR = 2.7; 95% CI: 1.02, 6.92) compared to those who were not.

The study participants living in the houses with poor ventilation were at risk of getting TB 4.0 times more than likely to develop tuberculosis (AOR = 4.02; 95% CI: 1.38, 11.76) compared to their counterparts. Similarly, the likelihood of having TB was about 3 times higher among the contacts dwelling in inadequate room size (less than 4 × 4 m per person) (AOR = 3.4; 95% CI: 1.30, 8.867) when compared to those who were living in an adequately sized room (Table 4).

## 4. Discussion

The prevalence of TB among adult household contacts of PTB patients was 7.8% (95% CI: 5.8–10.0). Frequency of meals per day, ventilation status of houses, sizes of living rooms, drinking raw milk, and family history of TB other than the index case, were identified as the factors significantly associated with tuberculosis.

The finding is comparable with the other studies in sub-Saharan Africa (7.8%) [18], Peshawar (0.1–14%) [19], and Tanzania (6.4%) [20], but, higher than that reported from Nepal (1.6%) [21], India (1.15%) [7], Pondicherry of India (4.3%) [22], South Africa (3.9%) [23], and Ethiopia (1%) [24]. The variation might be related to differences in the study population, living situation, and overcrowding which are important risks for respiratory diseases, including tuberculosis [25]. Differently, it is lower than the finding reported in a few of the previous studies conducted in Peru (34%) [26], Philippines (12.8%) [27], and Pakistan (15.6%) [28]. This difference could be associated with sample size of the study and diagnostic methods [1].

This study revealed that the likelihood of TB among household contacts eating meals less than three times per day was 4 times higher than that of individuals who eat meals three times or more per day. This is in line with the studies conducted in Tanzania [20] and Pakistan [29]. This might be due to undernutrition or nutritional deficiency that impairs cell-mediated immunity which can lead to progression of latent infection to active TB disease. The differences in the socioeconomic status, lifestyle, and feeding patterns of the household contacts might also explain these differences [30, 31].

Our study showed that household contacts who drink raw milk were 4 times more likely to develop TB compared to their counterparts. This is comparable with the studies conducted in Tanzania [32] and Gojam district of Ethiopia [15]. This might be due to intake of infected milk with the bacteria and which could disseminate from the initial location in the abdomen to other parts of the body via the blood stream, the lymphatic system, or by direct extension to other organs, which can be explained by the high prevalence of Bovine TB in the country [33, 34], and particularly in the study areas.

The likelihood of TB among household contacts with family history of TB other than index case were 8 times higher than that of individuals who did not have family history of TB (AOR = 2.78 CI: 1.01–6.92). This is in line with the studies done in India [10], Metema and Gojam in Ethiopia [15]. It is also in support of the WHO report which stated that there was greater occurrence of TB when the contact lived with more than one TB case [9]. It might be due to increased expelled bacilli that maximize the exposure within households.

The study finding showed that contacts dwelling in the house with poor ventilation were 4.0 times more likely at risk of getting TB than those dwelling in the house with good ventilation. This is consistent with the findings of other studies done in Pakistan [34], India [29], meta-analysis in sub-Saharan Africa [35], Addis Ababa, Ethiopia [36], Metema district, Ethiopia [16], and Gojam, Ethiopia [15]. This might be due to poor house ventilation that could increase the likelihood of exposure to tuberculosis by increasing the concentration of TB bacteria within households. However, good ventilation reduces the concentration of TB bacteria so it will decrease the transmission of the TB [37].

Household contacts living in inadequate sizes of rooms were 3 times more likely to get TB than those living in adequate sizes of rooms. This is in agreement with other studies done in sub-Saharan Africa [35], Pakistan [29], and Metema district of Ethiopia [16].This might be related with enclosed spaces and poor air circulation that can accelerate the transmission of TB [37].

Majority of socio-demographic factors assessed in this study did not show a statically significant association with the presence of TB in household contacts. However, community-based study done in high burden settings in Ethiopia [15] and Philippines [27] showed significant association of older age groups with the presence of TB. Similarly, occupational status of the household contacts were not significantly associated with the occurrence of TB in this study which is inconsistent with the study conducted in West Ethiopia [17]. This might be due to difference with study participants, lifestyle, and socioeconomic factors.

This study was not performed without limitations. Causal inferences (temporal relationship) cannot be drawn out since the study is a cross-sectional study. Smear negative cases were not verified using gold standard technique. During observation, there might be intra-observer variations. However, proper training was given to data collectors to minimize these biases. Therefore, this study could provide insights about the prevalence and associated factors of with TB disease among adult household contacts of PTB patients.

## 5. Conclusion

The overall prevalence of tuberculosis among adult household contacts of pulmonary tuberculosis patients is high. Eating meals less than three times per day, drinking raw milk, having family history of TB other than index case, living in poorly ventilated houses, and inadequate sizes in the living rooms were the independent predictors of the outcome. Appropriate health intervention should be given to the community by health extension workers on TB disease transmission, adequate and balanced diet, and safe dealing with diseased family member. Health facilities should screen all contacts of PTB patients as early as possible to reduce the number of active TB cases, and appropriate prompt treatment should be given to minimize the transmission. Health care providers should strengthen the information given to index cases that their close contacts and perform screening especially to those having the symptoms of TB (coughing, weight loss, and fever) and treat if required.

## Figures and Tables

**Table 1:** Socio-demographic characteristics of adult household contacts of smear positive PTB patients in Haramaya district, Eastern Ethiopia, 2019 (*n* = 451).

Variables		Frequency	
		Number	Percent (%)
Age (year)	18–27	212	47.0
	28–37	116	25.7
	≥38	123	27.3

Gender	Male	223	49.4
	Female	228	50.6

Marital status	Single	156	34.6
	Married	271	60.1
	Divorced	16	3.5
	Widowed	8	1.8

Ethnicity	Oromo	435	96.5
	Amhara	11	2.4
	Others	5	1.1

Religion	Muslim	441	97.8
	Orthodox	10	2.2

Residence	Urban	118	26.2
	Rural	333	73.8

Educational level	Not read and write	258	57.2
	Read and write	73	16.2
	Primary (1–8)	83	18.4
	Secondary (9–12)	25	5.5
	Collage and above	12	2.7

Occupation	Farmer	338	74.9
	Government employ	5	1.1
	House wife	37	8.2
	Merchant	14	3.1
	Daily labor	15	3.3
	Student	42	9.3

Relationship to the index case	Husband	112	24.8
	Wife	105	23.3
	Sibling	176	39.0
	Others	58	12.9

Monthly income of the family	<1000	273	60.5
	1000–1500	67	14.9
	>1500	111	24.6

**Table 2:** Housing condition of adult household contacts of smear positive PTB patients in Haramaya district, Eastern Ethiopia, 2019 (*n* = 451).

Variables		Frequency	
		Number	Percent (%)
Composition of house floor	Mud	418	92.7
	Cement & other	33	7.3

House ventilation status	Good	305	67.6
	Poor	146	32.4

Lighting status	Good	314	69.6
	Poor	137	30.4

Overcrowded	Yes	167	37.0
	No	284	63.0

Cooking energy	Electric	20	4.4
	Wood	408	90.5
	Others	23	5.1

Separated kitchen room	Yes	366	81.2
	No	85	18.8

Duration of index cases registered for treatment	≤6 month	125	27.7
	>6 month	326	72.3

Proximity to TB index case	Sleep on the same bed	217	48.1
	Sleep on different bed	234	51.9

Size of living room	Inadequate	148	32.8
	Adequate	303	67.2

**Table 3:** Behavioral related characteristics of adult household contacts of smear positive PTB patients in Haramaya district, Eastern Ethiopia, 2019 (*n* = 451).

Host (behavioral) related variables		Frequency (%)	
		Number	Percent (%)
Drinking alcohol	Yes	12	2.7
	No	439	97.3

Cigarette smoking	Yes	109	24.2
	No	342	75.8

Chewing khat	Yes	206	45.7
	No	245	54.3

Drinking raw milk	Yes	104	23.1
	No	347	76.9

Frequency of meals per day	≥3 meals per day	389	86.3
	<3 meals per day	62	13.7

Previously treated for TB	Yes	53	11.8
	No	398	88.2

Family history of TB other than index case	Yes	146	32.4
	No	305	67.6

**Table 4:** Factors associated with Tuberculosis disease among adult household contacts of smear positive PTB patients in Haramaya district, Eastern Ethiopia, 2019 (*n* = 451).

Variables	Category	Tuberculosis		COR (95% CI)	*p*-value	AOR (95% CI)	*p*-value
		Yes	No				
Age (year)	18–27	13 (6%)	199 (94%)	1		1	
	28–37	15 (13%)	99 (87%)	2.3 (1.06–5.06)	0.04	1.9 (0.55–6.87)	0.31
	≥38	7 (6%)	118 (94%)	0.9 (0.35–2.34)	0.84	0.3 (0.07–1.22)	0.09

Monthly income of the family	<1000	29 (11%)	244 (89%)	13.1 (1.76–97.20)	0.01	6.5 (0.63–68.38)	0.12
	1000–1500	5 (7%)	62 (93%)	8.9 (1.01–77.65)	0.05	15.1 (1.06–217.09)	0.06
	>1500	1 (1%)	110 (99%)	1		1	

House ventilation status^∗∗^	Poor	29 (19%)	122 (81%)	11.7 (4.71–28.77)	0.01	4.0 (1.382–11.76)	0.01
	Good	6 (2%)	294 (98%)	1		1	

Lighting status	Poor	22 (16%)	115 (84%)	4.4 (2.16–9.09)	0.01	0.78 (0.20–3.09)	0.72
	Good	13 (4%)	301 (96%)	1		1	

Overcrowded	Yes	23 (14%)	144 (86%)	3.6 (1.75–7.49)	0.01	1.3 (0.47–3.44)	0.63
	No	12 (4%)	272 (96%)	1		1	

Separated kitchen	Yes	18 (5%)	348 (95%)	1		1	0.53
	No	17 (20%)	68 (80%)	4.8 (2.37–9.85)	0.01	1.4 (0.47–4.40)	

Size of living room^∗^	Inadequate	24 (16%)	124 (84%)	5.1 (2.44–10.81)	0.01	3.4 (1.30–8.86)	0.01
	Adequate	11 (4%)	292 (96%)	1		1	

Proximity to index case	Sleep on the same bed	19 (9%)	198 (91%)	8.5 (4.12–17.59)	0.01	1.9 (0.61–6.32)	0.26
	Sleep on the different bed	16 (7%)	218 (93%)	1		1	

Treatment duration of index case	≤6 month	15 (12%)	110 (88%)	2.1 (1.03-4.22)	0.04	1.3 (0.46–3.41)	0.66
	>6 month	20 (6%)	306 (94%)	1		1	

Family Hx of TB other than index case^∗^	Yes	22 (15%)	124 (85%)	4.0 (1.95–8.16)	0.01	2.7 (1.02–6.92)	0.047
	No	13 (4%)	292 (96%)	1		1	

Smoking cigarette	Yes	18 (17%)	91 (83%)	3.9 (1.87–7.63)	0.01	1.4 (0.50–3.86)	0.54
	No	17 (5%)	325 (95%)	1		1	

Chewing khat	Yes	28 (14%)	178 (86%)	5.4 (2.28–12.52)	0.01	2.7 (0.91–7.74)	0.07
	No	7 (3%)	238 (97%)	1		1	

Drinking raw milk^∗∗^	Yes	27 (26%)	77 (74%)	14.9 (6.50–33.97)	0.01	4.1 (1.43–11.90)	0.01
	No	8 (2%)	339 (98%)	1		1	

Frequency of meals per day^∗∗^	<3 meals per day	21 (34%)	41 (66%)	13.7 (6.49–29.02)	0.01	4.3 (1.61–11.55)	0.004
	≥3 meals per day	14 (4%)	375 (96%)	1		1	

Overall knowledge scored on TB case	Inadequate	21 (24%)	68 (77%)	7.7 (3.72–15.84)	0.01	2.0 (0.72–5.54)	0.18
	Adequate	14 (4%)	348 (96%)	1		1	

^∗^Shows association having *p* value below 0.05 and ^∗∗^association having *p* value below 0.01. OR = odd ratio, COR = crude odd ratio, AOR = adjusted odd ratio, CI = confidence interval.

## Data Availability

All the necessary data supporting our findings are contained within the manuscript.
